# Systemic citrate load during continuous renal replacement therapy is not negligible and can be predicted using indirect methods

**DOI:** 10.1186/cc9549

**Published:** 2011-03-11

**Authors:** M Zakharchenko, M Balik, M Otahal, J Hruby, J Vavrova, A Jabor

**Affiliations:** 1 First Faculty of Medicine Charles University and General University Hospital, Prague, Czech Republic; 2 University Hospital, Hradec Kralove, Czech Republic; 3 IKEM, Prague, Czech Republic

## Introduction

Data on significance of systemic gain of citrate during continuous renal replacement therapy (CRRT) are missing. Direct citrate measurements are scarcely available. The quantification using a difference of unmeasured anions (UA) on the filter and the method using correlation between concentration of citrate (Cf) in effluent to the proportion of citrate flow to blood flow (Qc/Qb) were compared with the control exact methods.

## Methods

A prospective controlled observational study was performed in a 20-bed general ICU. Patients on 2.2% acid-citrate-dextrose (ACD, *n *= 41) were compared with controls on unfractioned heparin (*n *= 17). All were treated with an Aquarius Baxter device on 1.9 m^2 ^polysulfone filters. Samples were taken from a central venous catheter, ports pre filter and post filter and from dialysate/filtrate 24 hours after commencing with CRRT and 60 minutes later.

## Results

There were no significant differences (*P *> 0.05) between CVVH (*n *= 18) and CVVHDF (*n *= 23) in measured citratemias nor in systemic gain of citrate. The difference between post-filter and pre-filter UA correlated with difference of citrate concentrations (*r*^2 ^= 0.66). Citrate gain was calculated as 31.5 ± 10.5 mmol/hour utilizing this relationship. Cf showed tight correlation with the Qc/Qb ratio (*r*^2 ^= 0.72). Gain of citrate calculated as citrate input minus citrate removal (effluent flow × Cf) where the regression equation replaces Cf was 29.4 ± 7.2 mmol/hour. The first exact method used post-filter and pre-filter citrate concentrations multiplied by matching blood flows. Gain of citrate obtained by this method was 29.3 ± 11.0 mmol/hour. The second exact method deducted citrate removal (15.7 ± 5.9 mmol/hour) in effluent from citrate input (45.1 ± 8.8 mmol/hour) and produced a citrate gain of 29.3 ± 7.2 mmol/hour. Comparing two studied methods of citrate gain estimation with exact methods showed no significant differences (*P *= 0.5, Kruskal-Wallis ANOVA). Bland-Altman analysis showed no systematic bias in results.

## Conclusions

Systemic load of citrate is not negligible and can be predicted without taking direct citrate levels. Proposed indirect methods showed reasonable accuracy in systemic citrate load estimation.

